# Use of Smartphone-Based Video Directly Observed Therapy to Increase Tuberculosis Medication Adherence: An Interventional Study

**DOI:** 10.31661/gmj.v12i.3067

**Published:** 2023-07-03

**Authors:** Manal M. Al Daajani, Abdullah J. Alsahafi, Abdullah M. Algarni, Abdulhamed L. Moawwad, Ahmed A. Osman, Khalid Y.A. Algaali, Mohammed Abdalaziz, Muhammad A. Halwani, Shrooq M. Aldajani, Nazik M. H. Mohammed, Heassah S. Alshamrani, Mohammed N. Alshahrani, Ghadah M. Albostani, Naif G. Alshammari, Rami S. Alzahrani, Saadiya O. Alsomali, Ibrahim Assiri

**Affiliations:** ^1^ Public Health Department, Jeddah Health Affairs, Kingdom of Saudi Arabia (KSA); ^2^ Faculty of Medicine, Kassala University, Kassala, Sudan; ^3^ Public Health Operations Center, Ministry of Health, Riyadh, KSA; ^4^ Infection Prevention and Control Department, King Abdulaziz Medical City Ministry of National Guard, Jeddah, KSA; ^5^ Department of Microbiology, Faculty of Medicine, Al Baha University, Al Baha, KSA; ^6^ Department of Oral and Maxillofacial Sciences, Vision (Al-Farabi) Colleges for Dentistry and Nursing, Jeddah, KSA; ^7^ Faculty of Public Health and Health Informatics, Umm Al-Qura University, Makkah, KSA

**Keywords:** MDR-TB, Medication Adherence, Saudi Arabia, Smartphone, Tuberculosis, VDOT

## Abstract

Background: Tuberculosis (TB) treatment through Directly Observed Therapy (DOT) has an alternative form of video surveillance therapy (VOT) that utilizes the technological capabilities of smartphones to provide patients with low-cost access to doctors without impacting their work and personal life. We aimed to assess TB patients’ drug compliance, perceptions, and feasibility towards smartphone-based video direct observed therapy (VDOT) in Jeddah, KSA. Materials and Methods: We conducted a prospective non-randomized interventional study. We delivered smartphone-based VDOT among previously unstudied patients to monitor adherence to the treatment regimen. The expected total number of VDOT sessions was1200. We conducted post-intervention interviews to assess acceptability and satisfaction. Results: In this study, we included 20 participants, 16 of whom were males, with a mean age of 34.3 (±12.5) years. No side effects to the treatments were identified in all participants. The adherence rate for the total period was 93% and 99.5%, measured by the first and second methods, respectively. Most participants were satisfied with the VDOT experience, the time spent on sessions, and the approach’s privacy. Conclusion: This study provides promising results for the feasibility and effectiveness of smartphone-based VDOT for TB treatment to increase adherence which was indicated by a high compliance rate, acceptability, and high satisfaction level.

## Introduction

World Health Organization (WHO) reported that over 10 million people worldwide develop tuberculosis (TB) annually. The vision for the post-2015 global tuberculosis strategy is "a world without tuberculosis," also expressed as "zero deaths, disease, and suffering due to tuberculosis." Long-term TB treatment for at least six months results in drug discontinuation with a risk of developing drug resistance, disease persistence, death, and continued transmission of TB in the community [1]. In several infectious diseases including TB, human immunodeficiency virus (HIV), and hepatitis C, medication compliance is a significant factor leading to poor patient outcomes and promoting the development of drug-resistant tuberculosis (MDR-TB) and the spread of the disease [2]. Part of In-person-Directly Observed Therapy (DOT), an observation made three to five times a week at home, in the community, or the clinic, and it has been the standard of tuberculosis care for addressing the problem of poor adherence since the early 1990s published by WHO [1][3]. However, this approach carries several limitations, including inconvenience for patients and healthcare providers [3][4]. Additionally, DOT relies heavily on interpersonal interactions for supervision and support, which presents several challenges, particularly in resource-limited settings and in patients who are geographically distant and difficult to reach [5].

WHO has recommended video-observed therapy (VOT), which is daily remote monitoring using a smartphone application and is an alternative to in-person DOT by 2017 [3]. Video directly observed therapy (VDOT) studies in high, middle, and low-income countries indicate that patients adhered to their treatment regimens and reported high satisfaction levels despite evidence associated with individual DOT and tuberculosis treatment. have been shown to achieve and often prefer VDOT. Additionally, VDOT saves money for TB programs by reducing travel and staff costs [6][7][8][9]. Studies have looked at VOT alternatives that have less impact on patient’s work and family lives, more cost-effective, and improve patients’ access to doctors by utilizing the technological capabilities of smartphones [10][11][12]. Patient-centered care increased as the patients received VDOT as an option, necessitating ongoing communication, negotiation, and cooperation between patients and healthcare professionals [13].

With a funding gap (1.6 billion US dollars) in TB treatment in low- and middle-income countries, VDOT may be used to effectively allocate medical resources [14]. Through pilot studies collecting cost data, saving from the use of electronic DOT (eDOT) ranged from $1.811 to $14.355 per patient [15]. The total number of new and recurrent tuberculosis cases in the Kingdom of Saudi Arabia (KSA) in 2019 was 3004 cases, with an incidence of (8.7) per 100,000 and a therapeutic success rate of (89.9%). A private DOT program was successfully implemented in Jeddah. Subsequently, the Ministry of Health (MOH) expanded it to include two more provinces (Riyadh and Gazan districts) [16]. The use of VDOT to treat TB patients has been explored and considered by several prevention programs, especially after the Coronavirus disease (COVID-19) pandemic and lockdown. In this study, we aimed to assess the feasibility and perceptions of smartphone-based video communication to support adherence to tuberculosis medication among patients in Jeddah Province, KSA.

## Materials and Methods

The current study was a prospective single-arm non-randomized interventional study conducted in the National Tuberculosis Program (NTP) in the Public Health Department, Jeddah Health Affairs, KSA, from September to November 2021. We retrieved participants’ data from the three health centers that provided TB treatment. Participants were chosen based on inclusion criteria, which included stable patients aged 18 years or older with the drug-susceptible active pulmonary or extrapulmonary disease, patients receiving first-line anti-tuberculosis drug therapy administered only once daily orally with a fixed-dose combination (FDC) pill at an outpatient facility, and patients who discussed VDOT with them and voluntarily provided informed consent to allow their data to be used in future research. The exclusion criteria comprised any patients with MDR-TB or HIV, patients with other health conditions that could interfere with VDOT performance, including mental illnesses, impaired vision, hearing, or speech issues, patients who take pills more frequently than once per day, patients with drug contradictions or allergies, and patients who are incarcerated.

Based on practical considerations of recruitment viability and resource availability, we chose a small sample size of 20 participants because we believed that this would be a pilot study exploring the patients’ experience with the VDOT system. We used a quota sampling technique. The NTP staff invited eligible patients sequentially during DOT visits at home to enroll who met inclusion and exclusion criteria and consented to participate in the study.

Intervention

The investigators were provided with VDOT by a team trained by the Jeddah NTP. Research staff explained VDOT to the patients, asked them if they were interested and willing to participate, and obtained written informed consent. After that, we trained the staff and participants to use online video call meetings on their smartphones. By conducting a direct synchronized live video to observe participants, we measured adherence rate by asking the participants about their experience with drug intake and adverse events. Both staff and patients agreed on a regular pre-scheduled time set for all days of the week, and we modified it if the patient’s preference changed during treatment. We used the patient’s mobile phones to remind them of appointments and send them pill reminder notifications (via SMS) 30 minutes before each scheduled pill time. For two months, we followed them closely on the virtual platforms. If a patient misses three appointments, one of the research teams contacts them to resolve the concern. Once the researcher recognized the participant’s non-compliance, they were counseled, and a change to the scheduled pill time was suggested, as this might solve the underlying issue. At the end of the study period, the pills were counted after participants returned the pill bottles.

Outcome

We evaluated the adherence outcome in this single-arm interventional study. All analyses were based on the two months of follow-up. The total expected VDOT sessions were 60 sessions per participant.

Treatment Adherence

We determined the adherence for each patient in two ways: first, by applying the equation [(videos observation confirming pill intake plus videos not received due to technical problems)/videos expected] ×100 [4]. After that, we measured adherence by pill count, an indirect objective method, to confirm the result and minimize bias. Compliance was calculated for each patient based on video sessions outcome using the following formulae:

Adherence=(videos with observed dose intake-videos missed)/ (total expected videos -videos with suspended intake)×100 [4].

Confirmed adherence=[(OT+ONT) - (M+R&M)]/(OT+ONT+M+R&M-S)×100 [4].

Assumed adherence=[(OT+ONT+R&M) -(M)]/(OT+ONT+M+R&M-S)×100 [4].

OT=Doses observed on time. ONT=Dose observed but not on time. M=Dose missed. R&M=Received and Missed Dose in the video. S=Suspension due to medical advice.

We used an interview questionnaire as a data collection tool including age, sex, socioeconomic characteristics, education, occupation, relevant medical information (comorbidities), VDOT sessions, and drug side effects. After completion of the intervention (two-month treatment period), we assessed feasibility, acceptability, satisfaction rate, and general perception among participants in a post-study survey.

Data Analysis

We collected and entered data into Statistical Package for Social Sciences (SPSS) version 22 (IBM Corporation, Armonk, NY, USA). We conducted the statistical analysis using continuous and categorical variables. We presented the continuous variables as a range, whereas categorical were presented as numbers and percentages. We calculated frequencies and means ± standard deviation (SD) to summarize continuous variables. We applied the Chi-square test in inferential statistics and considered differences in the results statistically significant with a two-sided P≤0.05 and 95% confidence interval (CI).

Ethical Considerations

Informed consent was obtained from the patients with a clear explanation of the purposes of this study and they were able to terminate their voluntary contribution at any time without affecting their treatment Institutional review board (IRB) approval was obtained from Jeddah Health Affairs, KSA number: H-02-J-002, Research No. 1544.

## Results

**Table:** Table1. The Sociodemographic Characteristics of the Participants (N=20).

Characteristic		Count (%)
Sex	Male	16 (80%)
	Female	4 (20%)
Age (in years)	The mean age 34.3 (±12.5) years	
	10-19	2 (10%)
	20-29	9 (45%)
	30-39	1 (5%)
	40-49	4 (20%)
	50 years and above	4 (20%)
Education	Illiterate	4 (20%)
	Primary school	1 (5%)
	Middle school	1 (5%)
	Secondary school	8 (40%)
	University & above	6 (30%)
Occupation	Employed	11 (55%)
	Student	6 (30%)
	Unemployed	3 (15%)
Monthly income in SAR*	Less than 3,000	16 (80%)
	3,000 - 5,999	4 (20%)

^*^ **SAR:** Saudi Arabian Rial

**Table:** Table2. VDOT Adherence (N=20).

VDOT sessions		Month 1	Month 2	Total period	p Value
Duration (in minutes)	Mean (±SD)	7.4 (±3.1)	5 (±3.4)	6.2 (±3.5)	0.69
	Median	5	4	5
	Range	1-16	1-15	1-16
Outcome	OT*	554 (92%)	559 (93%)	1113 (93%)	0.68
	ONT**	43 (7%)	38 (6%)	81 (7%)
	M***	2 (0%)	3 (1%)	5 (0%)
	R&M†	1 (0%)	0 (0%)	1 (0%)
	S††	0 (0%)	0 (0%)	0 (0%)
	Total	600 (100%)	600 (100%)	1200 (100%)
Adherence	Confirmed	99.5%	99.5%	99.5%	0.99
	Assumed	99.7%	99.5%	99.6%	0.99

^*^ **OT:** Doses observed on time; ^**^ **ONT:** Dose observed but not on time; ^***^ **M:** Dose missed; **† R&M:** Received and Missed dose in the video; **†† S:** Suspension due to medical advice.

**Table:** Table3. The Satisfaction Level, Privacy, and Easiness VDOT According to Participants’ Perception (N=20).

	Satisfaction level	Count (%)
General satisfaction level	Satisfied	16 (80%)
	Dissatisfied	4 (20%)
Satisfaction with time spent on VDOT sessions	Very satisfied	16 (80%)
	Satisfied	4 (20%)
Privacy on VDOT vs. in-person DOT	Higher	16 (80%)
	Same	2 (10%)
	Lower	2 (10%)
Overall VDOT process easiness	Very easy	15 (75%)
	Somewhat easy	5 (25%)
	Somewhat or very difficult	0 (0%)

**Table:** Table4. VDOT Sessions Issues and Frequency of Taking the Teatment Outside the House (N=20).

Sessions issues		Count (%)
VDOT sessions issues	Network connectivity	5 (25%)
	Internet subscription	3 (15%)
	Rare	8 (40%)
	Never	4 (20%)
Frequency of taking the treatment outside the house	Most the times	8 (40%)
	Rare or never	12 (60%)

**Table:** Table5. Participants’ Reasons to Continue VDOT

Reasons to continue VDOT	Descriptions	Count (%)
Easier to use	Easiness of use	8 (40%)
Mobility	Ability to conduct VDOT session from anywhere	5 (25%)
Easier communication	Ease of communication with the team	4 (20%)
Lower cost/effort	Saving time and effort of attending the clinic	3 (15%)
Privacy	Higher privacy	1 (5%)

**Table:** Table6. Participants’ Perception about Using VDOT (N=20).

Participants’ perception		Count (%)
If re-treatment is required, what would you choose?	VDOT	20 (100%)
	DOT	0 (0%)
Recommend VDOT to other TB patients	Yes	20 (100%)
	No	0 (0%)

In this study, we included 20 participants with a median age of 34.3 (±12.5) years (18 - 54 years). Eighty percent of them were males, and 40% of them had a secondary school education. More than half of the participants were employed, and about 80% had a monthly income of less than 3000 Saudi Arabian Rials (SAR) (Table-1). The total expected VDOT sessions were 1200 sessions.

Over the course of the study, we haven’t identified any side effects to the treatments in all participants that could lead to treatment discontinuation. The mean duration (in minutes) was 6.2 (±3.5) for the total period. More than 90% of participants were found to adhere to most doses on time, suggesting a 93% overall adherence rate as measured by the first method.

Moreover, the adherence rate for the second method reached (99.5%). There are no statistically significant differences between the first and second months in the duration, outcome, and adherence (Table-2). According to the prescribed management method, the post-VDOT survey revealed the participants’ satisfaction level with the VDOT experience. We found that four-fifths of the participants were satisfied with their VDOT experience, the time spent on VDOT sessions, and their privacy, while only one-fifth were dissatisfied with the VDOT experience.

The overall VDOT process feasibility was described as very easy by three-quarters of the participants (Table-3). We observed that some participants experiences VDOT session issues, for example a quarter had poor internet connection and 15% had an internet subscription. At the same time, two-fifth of them rarely had VDOT session issues or one-fifth had none at all.

We noticed that a considerable number of participants (40%) took their sessions outside their houses (Table-4). In this study, the participants had several reasons to continue VDOT in the future if it becomes available. One of these reasons is the ease of use, reported by 40% of participants, and the ability to conduct VDOT sessions from anywhere, which was reported by one-quarter of the participants (Table-5).

Generally, the participants’ perceptions about using and recommending VDOT to other patients were 100% positive, as shown in (Table-6).

## Discussion

In this study, we included a total of 20 participants who used smartphone-based video call for conducting VDOT and were followed for two months with a total of 1200 sessions. The study found a high level of satisfaction (80%) among participants and a very high level of medication regimen adherence (99.5%) using the smartphone-based VDOT method. In KSA, the non-compliance rate of DOT reaches 28% with an adherence rate of 72% [17].

Usually, the completion of 80% or more of scheduled treatment observations is considered a success and the optimum rate of DOT [18][19]. In comparison, the suboptimum level of DOT is considered below 75% of completion of the treatment regimen [20]. A pilot project conducted in India revealed that the overall adherence rate of VDOT was 96.03%, the average adherence rate by call was 92.25%, and the average adherence rate by in-person DOT (standard DOT) was 32.12% [21].

The results of this study are consistent with other studies on VDOT, which have also found high levels of adherence and patient satisfaction. For example, a survey conducted in the United States aimed to assess the feasibility and acceptability of VDOT. It was a single-arm trial among TB patients, recruiting 52 patients (from San Diego and Tijuana, USA) with a mean age of 37 years; half were male. The reported adherence rate with vDOT was (93%) in San Diego and (96%) in Tijuana [22]. Similarly, in India, Holzman et al. found that VDOT was a feasible and effective method of TB treatment, with high levels of adherence and patient satisfaction [23].

However, in this study, we found that some participants reported some issues and difficulties, including network connection and internet subscription, as well as using the Facetime application. This lack of resources was a potential limitation of the method that should be addressed for the future effective implementation of VDOT. Alternative video platforms or training programs may need to be considered to improve patient ease of use.

A study conducted in Ethiopia found that VDOT was a feasible and effective method of TB treatment, with high levels of adherence and patient satisfaction. However, the study also found that patients preferred in-person DOT over VDOT, which may reflect cultural differences in patient preferences and expectations [24]. Our findings are similar to other results of previous studies conducted in South Africa, which found that VDOT was a feasible method of TB treatment, but also identified concerns about privacy and confidentiality among patients. This concern has been identified in other studies and highlights the importance of addressing patient concerns and ensuring the security of patient information while using VDOT [25]. We noticed that many participants felt comfortable using VDOT in their houses or outside, which added value to using VDOT versus in-person DOT. Overall, these studies suggest that VDOT is a promising new method for TB treatment that can improve adherence to medication regimens and reduce the cost of TB treatment. Further research is needed to fully understand the potential benefits and drawbacks of VDOT and identify the best practices for its implementation in TB treatment programs, taking into account cultural and contextual factors that may affect its feasibility and acceptability.

## Conclusion

In conclusion, this study strongly conveys the feasibility and effectiveness of smartphone-based VDOT for TB treatment in increasing compliance, as evidenced by a high adherence rate, acceptability, and high satisfaction level among participants.

## Conflict of Interest

The authors declare no conflict of interest.

## References

[R1] Global Tuberculosis Report 2020.: World Health Organization..

[R2] Martin LR, Williams SL, Haskard KB, DiMatteo MR (2005). The challenge of patient adherence. Therapeutics and clinical risk management.

[R3] Story A, Aldridge RW, Smith CM, Garber E, Hall J, Ferenando G, et al (2019). Smartphone-enabled video-observed versus directly observed
treatment for tuberculosis: a multicentre, analyst-blinded, randomised,
controlled superiority trial. The Lancet.

[R4] Molton JS, Pang Y, Wang Z, Qiu B, Wu P, Rahman-Shepherd A, et al (2016). Prospective single-arm interventional pilot study to assess a
smartphone-based system for measuring and supporting adherence to medication. BMJ open.

[R5] Rabinovich L, Molton JS, Ooi WT, Paton NI, Batra S, Yoong J (2020). Perceptions and acceptability of digital interventions among
tuberculosis patients in Cambodia: qualitative study of video-based directly
observed therapy. Journal of Medical Internet Research.

[R6] Mirsaeidi M, Farshidpour M, Banks-Tripp D, Hashmi S, Kujoth C, Schraufnagel D (2015). Video directly observed therapy for treatment of tuberculosis is
patient-oriented and cost-effective. European Respiratory Journal.

[R7] Chuck C, Robinson E, Macaraig M, Alexander M, Burzynski J (2016). Enhancing management of tuberculosis treatment with video
directly observed therapy in New York City. The International Journal of Tuberculosis and Lung Disease.

[R8] Hayward A, Garber E (2015). TB Reach 5: to compare the efficacy of video observed treatment (VOT) versus directly observed treatment (DOT) in supporting adherence in patients with active tuberculosis.

[R9] Skrahina A, Falzon D, Rusovich V, Zhylevich L, Dara M, Sinkou H, et al (2017). Video observed treatment for tuberculosis patients in Belarus. Eur Respiratory Soc.

[R10] Ingram D (2018). Video directly observed therapy: Enhancing care for patients with
active tuberculosis. Nursing2020.

[R11] Subbaraman R, de Mondesert, Musiimenta A, Pai M, Mayer KH, Thomas BE, et al (2018). Digital adherence technologies for the management of tuberculosis
therapy: mapping the landscape and research priorities. BMJ global health.

[R12] Macaraig M, Lobato MN, Pilote KM, Wegener D (132018). A national survey on the use of electronic directly observed
therapy for treatment of tuberculosis. Journal of Public Health Management and Practice.

[R13] Garfe15in RS, Liu L, Cuevas-Mota J, Collins K, Muñoz F, Catanzaro DG, et al (2018). Tuberculosis treatment monitoring by video directly observed
therapy in 5 health districts, California, USA. Emerging infectious diseases.

[R14] (2023). Global tuberculosis report 2022.

[R15] (October 04, 2019). Electronic Directly Observed Therapy for Active TB Disease.

[R16] National Tuberculosis Program Manual 2021.

[R17] Chaudhry LA (2015). Low rate of non-compliance to antituberculous therapy under the
banner of directly observed treatment short course (dots) strategy and well
organized retrieval system: a call for implementation of this strategy at
all dots centers in saudi arabia. The pan african medical journal.

[R18] Story A (2019). Smartphone-enabled video-observed versus directly observed
treatment for tuberculosis: a multicentre analyst-blinded randomised
controlled superiority trial. Lancet (london england).

[R19] Nahid P, Dorman SE, Alipanah N, et al (2016). Official American thoracic society centers for disease control
and prevention infectious diseases Society of America clinical practice
guidelines treatment of drug-susceptible tuberculosis. Clinical Infectious Diseases.

[R20] (2016). World Health Organization.

[R21] Thakkar D, Piparva KG, Lakkad SG (2019). A pilot project: 99DOTS information communication
technology-based approach for tuberculosis treatment in Rajkot district. Lung India.

[R22] Garfein RS, Collins K, Muñoz F, Moser K, Cerecer-Callu P, Raab F, Rios P, Flick A, Zúñiga ML, Cuevas-Mota J, Liang K, Rangel G, Burgos JL, Rodwell TC, Patrick K (2015). Feasibility of tuberculosis treatment monitoring by video
directly observed therapy: a binational pilot study. Int J Tuberc Lung Dis.

[R23] Holzman SB, Atre S, Sahasrabudhe T, Ambike S, Jagtap D, Sayyad Y, Kakrani AL, Gupta A, Mave V, Shah M (2019). Use of Smartphone-Based Video Directly Observed Therapy (VDOT) in
Tuberculosis Care: Single-Arm, Prospective Feasibility Study. JMIR Form Res.

[R24] Bommakanti KK, Smith LL, Liu L, et al (2020). Requiring smartphone ownership for mHealth interventions: who
could be left out. BMC Public Health.

[R25] Sekandi JN, Kasiita V, Onuoha NA, et al (2021). Stakeholders' Perceptions of Benefits of and Barriers to
Using Video-Observed Treatment for Monitoring Patients With Tuberculosis in
Uganda: Exploratory Qualitative Study. JMIR Mhealth Uhealth.

